# An Epitome of Certain Views on Epidemics or Contagion
*Translated from the German.


**Published:** 1855-12

**Authors:** 

**Affiliations:** formerly Residential Physician in the Prussian Army.


### AN EPITOME OF CERTAIN VIEWS ON EPIDEMICS OR CONTAGION.*

By Dr. Riecke, formerly Residential Physician in the Prussian Army.

I have the honour to present to the Epidemiological Society of London the five volumes of my contributions to State medicine, and venture to request that the Society will kindly accept and leniently judge of them.

I think that my writings show that I have worked in accordance with the spirit of the honourable Society, and I may regard myself therefore, in that sense, as having laboured to promote its objects.

The special titles and prefaces explain the contents and the purport of my writings. The first four volumes are, as it were, the propædiatics to the fifth volume. In the latter I have developed the following doctrines: —

1. That all contagia,† under certain circumstances, have a primary origin; and that even syphilis may be primarily produced. This is a fundamental principle.

2. That in this process the mucous membranes play the most important part: and especially that the volatile contagium is generated and secreted by them.

3. That the contagia differ in their effect, intensity, etc.; but that they are identical in certain respects.

4. That in the part which contagia play in epidemics, they only contribute to increase the number of seizures in the limited focus (nidus) of disease, but that they are unable to diffuse a malady over countries and nations.

---

* Translated from the German.

† Contagia—short for contagious matters; the principle of contagions.

5. That there is no such thing, and never has been, as a contagium capable of communication by the mere contact of the hand with the patient.

6. That epidemics arise in causes which are inherent in the terrestrial body. These produce the disposition in the living organism, and the so-called exciting causes (amongst which I count the contagium itself) induce the eruption of the malady. It is not until this stage that the contagium is generated by the morbid process.

7. That there is no contagium which is capable of spreading a disease within a few months over the globe,—not even the plague and the small-pox.

8. That the so-called miasmetic contagium arises in individuals disposed to it, if the predisposition is much developed. If many highly predisposed individuals are shut up in close, ill-ventilated rooms, the malady originates primarily among them; they, as it were, poison themselves. For this reason, persons living in such places, who are only slightly attacked, become dangerously affected; and the intensity of the contagium attains such a degree in such localities, that persons are affected whose predisposition is only slight.

---

